# A novel measure of treatment benefit for an ordinal scale: a case study of the IST-1 and the IST-3 stroke trials

**DOI:** 10.1186/1745-6215-14-S1-O48

**Published:** 2013-11-29

**Authors:** Douglas Thompson, William Whiteley, Gordon Murray

**Affiliations:** 1 Edinburgh MRC Hub for Trials Methodology Research, University of Edinburgh, Edinburgh, UK; 2 Division of Clinical Neurosciences, University of Edinburgh, Bramwell Dott Building, Western General Hospital, Edinburgh, UK

## Background

Relative measures quantify the effect of an intervention but are difficult to translate into practice. Clinicians prefer absolute measures like the number needed to treat (NNT). We demonstrate a novel approach for reporting treatment effect across an ordered outcome where higher scores indicate worse functional outcome. We used the first and the third International Stroke Trials (IST-1 and IST-3) as case studies.

## Methods

A two by ‘K' table of treatment versus control over an ordinal outcome (with K levels) can be modelled as a multinomial distribution and the probabilities for each cell estimated. We calculated the number of score points gained per 1000 patients treated and estimated the 95% CI using bootstrap methods. Negative values indicate benefit with treatment whilst positive values indicate harm with treatment. We categorised patients into groups of poor functional outcome using prediction models (low (≤35%), medium (35 to 56%), and high (>56%)) and calculated the net gain in functional outcome within each stratum.

## Results

The gain in Oxford Handicap Score (OHS) points per 1000 treated in IST-3 for low risk was 14 (95% CI -199 to 240), for medium risk -295 (95%CI -566 to -19) and for high risk -230 (95%CI -396 to -65). The gain in a four level functional outcome score in IST-1 for low risk was -112 (95% CI -214 to -9), for medium risk -42 (95% CI -95 to 12) and for high risk -29 (95% CI -59 to 2).

## Conclusions

A ‘net reduction in disability per 1000 patients treated' could be reported alongside the common odds ratio from the proportional odds model.

